# Immune Checkpoint Inhibitor-Induced Pneumonitis in Non-Small Cell Lung Cancer: A Narrative Review of Incidence and Clinical Risk Factors

**DOI:** 10.3390/curroncol33050240

**Published:** 2026-04-22

**Authors:** Olexiy Aseyev, Liliia Zrielykh, Minghan Shi, Katherine Filipovic, Claire Seymour, Rabail Siddiqui, Birubi Biman

**Affiliations:** 1 Regional Cancer Centre, Thunder Bay Regional Health Sciences Centre, 980 Oliver Road, Thunder Bay, ON P7B 6V4, Canada; 2 Division of Clinical Sciences, Northern Ontario School of Medicine University, 955 Oliver Road, Thunder Bay, ON P7B 5E1, Canada; 3 Polyclinic Department, Universal Clinic “Oberig”, 3 Zoolohichna Street, 03057 Kyiv, Ukraine; 4 Internal Medicine, Northern Ontario School of Medicine University, 955 Oliver Road, Thunder Bay, ON P7B 5E1, Canada; 5 Clinical Research Services, Thunder Bay Regional Health Research Institute, 980 Oliver Road, Thunder Bay, ON P7B 6V4, Canada; 6 Michael G. DeGroote School of Medicine, McMaster University, 1280 Main Street West, Hamilton, ON L8S 4L8, Canada; 7 Internal Medicine, Thunder Bay Regional Health Sciences Centre, 980 Oliver Road, Thunder Bay, ON P7B 6V4, Canada

**Keywords:** non-small cell lung cancer, pneumonitis, immunotherapy, immune checkpoint inhibitors

## Abstract

It is well-known that immune checkpoint inhibitors (ICIs) can lead to uncommon but potentially life-threatening immune-related adverse events (irAEs) such as checkpoint-inhibitor pneumonitis (CIP). Early recognition, identification, and treatment are crucial with regard to decreasing toxicity from ICIs. However, there is a discrepancy in the incidence rates between the clinical trials and postmarketing studies. Several postmarketing studies have developed early detection methods and identified new risk factors in developing CIP. Thus, in this narrative review, we aim to review these incidence rates, early detection methods, and clinical risk factors for CIP in NSCLC patients, which could help to improve CIP diagnosis and management for enhanced NSCLC care. Major clinical trials and postmarketing studies of CIP incidence, early detection methods, and risk factors in NSCLC were reviewed. A wide array of potential risk factors has been implicated in the development of CIP in NSCLC, such as having preexisting interstitial lung disease, receiving PD-1 inhibitors, receiving combination ICI therapy instead of ICI monotherapy, lower pretreatment hemoglobin and albumin levels, increased baseline plasma IL-8 levels, and impaired baseline pulmonary function.

## Simple Summary

Immunotherapy has become part of the main treatment for non-small cell lung cancer. However, it pertains with immunotherapy-related inflammation of the lungs, an uncommon but potentially life-threatening side effect. Identification of patients at risk, early detection, and treatment of this inflammation is crucial in improving symptoms and reducing death rates. In this article, we aim to find the real-life incidence rate of this side effect and review studies with risk factors and early detection methods to help in early recognition and treatment decision-making related to this uncommon but serious immunotherapy-related lung inflammation.

## 1. Introduction

Lung cancer is the most commonly diagnosed cancer worldwide and remains the leading cause of cancer mortality, making up 12.4% of all new cases and 18.7% of cancer deaths [1]. Non–small cell lung cancer (NSCLC) accounts for about 85% of all lung cancers [2]. The introduction of immune checkpoint inhibitors (ICIs) has significantly improved survival outcomes, including OS, progression-free survival (PFS), and event-free survival (EFS) in resectable, locally advanced, and metastatic NSCLC [3,4,5,6,7].

ICIs are monoclonal antibodies that enhance anti-tumor immune responses by blocking inhibitory immune checkpoint receptors on T-lymphocytes, thereby releasing the brakes on the immune system and revolutionizing lung cancer therapy [8]. Approved ICIs for NSCLC include inhibitors targeting programmed cell death-1 (PD-1, e.g., pembrolizumab), cytotoxic T-lymphocyte antigen-4 (CTLA-4, e.g., ipilimumab), and programmed death-ligand 1 (PD-L1, e.g., atezolizumab). However, by exciting immune activity, these agents can also disrupt normal immune tolerance mechanisms, leading to a spectrum of immune-related adverse events (irAEs) [9,10], including checkpoint-inhibitor pneumonitis (CIP) [11,12].

CIP is a rare but potentially life-threatening irAE characterized by immune-mediated focal or diffuse inflammation of the lung parenchyma following ICI therapy [13,14]. Clinically, CIP can present asymptomatically or with dyspnea and other respiratory symptoms (e.g., cough), accompanied by new inflammatory infiltrates on chest imaging after ICI initiation, in the absence of infection or tumor progression [15,16].

## 2. Incidence

According to clinical trial data, the incidence of CIP in NSCLC ranges from about 3% to 6.3%, with a reported mortality rate under 1% [17]. In real-world settings, however, higher pneumonitis rates have been observed, with approximately 5% to 19% of ICI-treated NSCLC patients developing CIP outside of trials [18,19] (Table 1). This discrepancy may be explained by broader patient inclusion in routine practice (e.g., patients with pre-existing lung conditions or poorer performance status who would be excluded from trials) [20]. Greater awareness and vigilance in recognizing CIP, as well as formal adjudication of pneumonitis cases in registries, have also likely contributed to higher reported incidences in recent years [20,21]. See Table 2 for the incidence of ICI-induced pneumonitis in NSCLC clinical trial data.

## 3. Early Detection Methods

Among all irAEs in NSCLC, CIP is particularly concerning due to its potential lethality. Development of moderate-to-severe CIP often requires prompt high-dose corticosteroid therapy (e.g., methylprednisolone) and sometimes additional immunosuppressive treatments (such as tocilizumab, infliximab, or cyclophosphamide) to control inflammation [11,43,44]. Severe CIP typically mandates permanent discontinuation of ICI therapy, and can still lead to respiratory failure or death despite treatment [11,14]. Indeed, although relatively uncommon, CIP is one of the leading causes of treatment-related fatality in NSCLC patients receiving ICIs [12,22].

Early recognition of CIP is critical because timely intervention, primarily with corticosteroids, can prevent progression to respiratory failure. Oncologists and pulmonologists should maintain a high index of suspicion for pneumonitis in any ICI-treated patient who develops new respiratory symptoms. Baseline and periodic pulmonary function tests (PFTs) and imaging are useful tools for monitoring patients at risk.

### 3.1. PFTs

Several studies suggest that patients who develop CIP tend to have lower pre-treatment lung function. For instance, a retrospective analysis found that NSCLC patients who developed pneumonitis had a lower baseline forced expiratory volume in 1 s (FEV_1_) % predicted compared to those who did not, although baseline forced vital capacity (FVC) was similar between the groups, despite a comparable prevalence of COPD [45]. Similarly, a prospective multicenter study by Suzuki et al. [46]. reported that impaired baseline spirometry (reduced FVC and FEV_1_) and the presence of baseline dyspnea symptoms (as measured by a standardized dyspnea scale) were associated with a higher incidence of immune-related pneumonitis in patients receiving ICIs [46].

### 3.2. Radiologic Evaluation

Radiologic evaluation is the cornerstone of early detection of CIP. Routine CT scans in patients receiving immunotherapy can incidentally detect asymptomatic pneumonitis changes, such as ground-glass opacities or organizing pneumonia patterns, before clinical symptoms worsen. Prompt recognition of these imaging patterns, along with their correlation to clinical findings, can facilitate early management. In practice, when CIP is suspected, it is essential to rule out infectious etiology and tumor progression (often via bronchoscopy with lavage and, in some cases, lung biopsy) to confirm the diagnosis [15,16].

Once CIP is diagnosed, management follows established guidelines based on severity. Grade 1 (mild, asymptomatic) pneumonitis may be managed with close monitoring and temporarily suspending ICI treatment, whereas Grade 2 or higher (moderate-to-severe) pneumonitis warrants immediate immunosuppressive therapy, such as high-dose corticosteroids, and discontinuation of immunotherapy. If there is no improvement within 48–72 h of initiating steroids or in steroid-refractory cases, additional immunosuppressants such as infliximab (anti-TNF), mycophenolate mofetil, or intravenous immunoglobulin (IVIG) may be considered per guideline recommendations [11,44].

### 3.3. Early Consultation and Multidisciplinary Discussion

Early pulmonology consultation and a multidisciplinary approach, including oncology, pulmonology, radiology, and infectious disease specialists, is recommended to optimize CIP diagnosis and management. Rapid intervention is crucial, as CIP, although relatively rare, can progress quickly and has been identified as a major cause of ICI-related deaths [12].

## 4. Clinical Risk Factors for Checkpoint-Inhibitor Pneumonitis

### 4.1. Pre-Existing Interstitial Lung Disease

**Figure 1:**
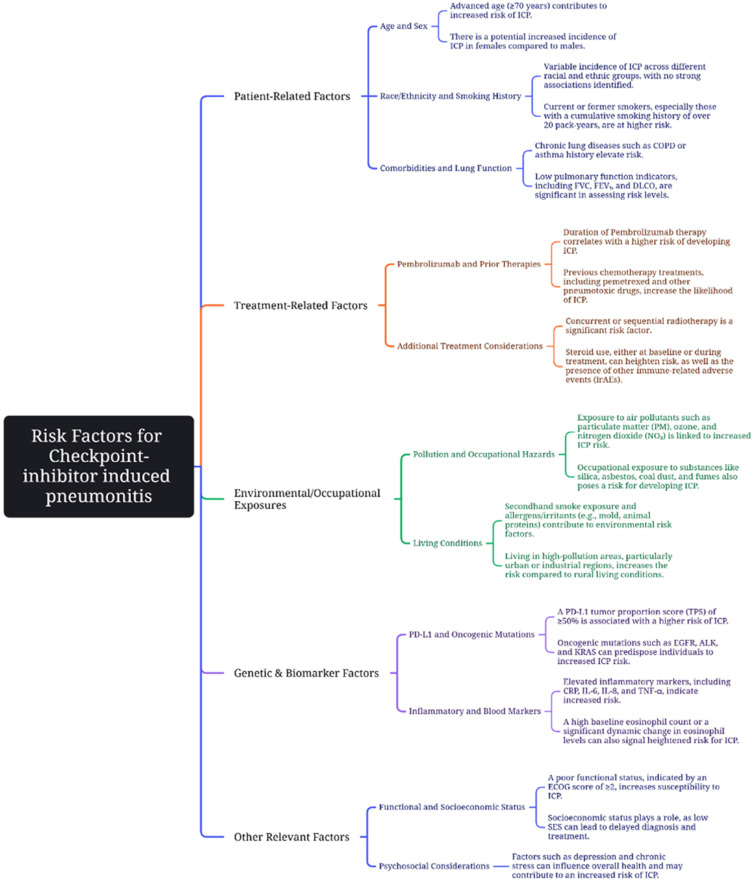
Risk factors contributing to ICP in immunotherapy. These risk factors include: advanced age [23,24,54]; female sex [20,22]; smoking history [22,23,24]; chronic lung disease [22,23,24,54]; low pulmonary function indicators [29]; duration of pembrolizumab therapy [20,21,25]; previous chemotherapy treatments [20,21,24]; concurrent or sequential chemoradiotherapy [10,23,24]; exposure to air pollutants [10]; elevated PD-L1 tumor proportion score [21,22]; elevated inflammatory markers [22]; eosinophil levels [29]; poor functional status [10,55]. Incidence of ICP has no strong associations with race or ethnicity [20] (This figure was created using Microsoft Visio Professional 2019).

Identifying risk factors predisposing patients to checkpoint-inhibitor pneumonitis (CIP) is of great importance, as it may allow for preventive strategies or closer monitoring in high-risk individuals. Some of these risk factors can be found outlined in Figure 1. One consistently reported risk factor for ICI-related pneumonitis is pre-existing interstitial lung disease (ILD). ILD has been reported to exist in approximately 15% of lung cancer patients at the time of initial diagnosis and is associated with a poor prognosis [28,47,48].

Patients with underlying ILD, such as idiopathic pulmonary fibrosis, are far more likely to develop CIP when treated with ICIs. In a retrospective study of NSCLC patients receiving combination chemo-immunotherapy, those with pre-existing ILD had an odds ratio (OR) of approximately 19 for developing treatment-related pneumonitis compared to patients without ILD [21]. The same study also found that patients receiving pemetrexed-based chemotherapy in combination with ICIs had a higher risk of pneumonitis, suggesting that certain cytotoxic agents (e.g., pemetrexed) might potentiate lung inflammation [20]. In a retrospective study of NSCLC conducted by Kanai et al. [49], the incidences of nivolumab-related pneumonitis and severe nivolumab-related pneumonitis were significantly higher in patients with pre-existing ILD, although the treatment outcomes for pneumonitis were comparable between patients with and without pre-existing ILD [49]. In a retrospective cohort study by Sawa et al. [26], the risk of developing pneumonitis with ICIs in NSCLC patients with pre-existing ILD was similar to that observed with conventional chemotherapy [26].

Altan et al. [24] demonstrated that ILD in non-smokers and a longer duration of smoking, along with baseline symptoms of shortness of breath or cough in smokers, are associated with a higher risk of pneumonitis. However, the ability to evaluate the etiology of baseline symptoms was limited by the paucity of pre-treatment pulmonary function data [24].

### 4.2. History of Lung Irradiation

An earlier analysis by Barrón et al. [10] reported that a history of thoracic radiotherapy was associated with an increased likelihood of CIP and worse overall survival with subsequent immunotherapy, indicating that prior lung irradiation is another important risk factor to consider [10].

### 4.3. Chronic Obstructive Pulmonary Disease (COPD)

The influence of smoking history and chronic lung diseases such as COPD on CIP risk is less clear. Although COPD is common in lung cancer patients (in newly diagnosed lung cancer patients, it is estimated to be about 50%) and contributes to baseline pulmonary impairment, one analysis by Zeng et al. [28] showed that coexisting COPD (as defined by spirometry) did not significantly increase the risk of pneumonitis in NSCLC patients treated with ICIs. In contrast, another retrospective report observed that approximately 70% of patients who developed CIP had a history of COPD; however, this study relied on physician-diagnosed COPD rather than pulmonary function criteria, potentially confounding the results [28]. Overall, factors such as smoking status, older age, and common comorbidities (e.g., diabetes) have not consistently shown significant associations with CIP in most studies [22,28]. For example, a large single-institution study by Liu et al. [23] found that age, tumor histology, smoking history, pre-existing lung disease, performance status, concurrent chemotherapy, and baseline blood counts (neutrophil-to-lymphocyte ratio or monocyte counts) were not significantly different between patients who developed CIP and those who did not, suggesting that these factors were not strong predictors in that cohort [23].

In the study by Chao et al., the presence of COPD was associated with a higher incidence of CIP, and COPD patients experienced a lower grade of CIP, mainly Grade 2 [22].

### 4.4. PD-L1 Expression

In the study by Chao et al., PD-L1 expression of ≥50% was associated with a higher incidence of CIP [22].

In meta-analyses of controlled clinical trials conducted by Kong et al. [15], it was shown that ICI use was associated with a higher risk of both all-grade (1–5) and high-grade (3–5) pneumonia compared to chemotherapy, regardless of histology, treatment regimen, PD-L1 expression level, negative EGFR/ALK expression, or previous treatment history. Subgroup analyses revealed that the squamous group, the ICI versus combination chemotherapy group, the PD-L1 >50% group, and the previously untreated group had a higher risk of developing both all-grade and Grade 3–5 CIP [15]. However, in the study by Cho et al. [50], the proportion of tumors with positive PD-L1 expression did not differ between patients with and without ICI-related pneumonitis (i.e., CIP). In this study, extrathoracic metastases were less frequent (31.8% versus 58.6%) in patients with pneumonitis, whereas previous ILD (OR = 6.03, 95% CI: 1.19–30.45) and the absence of extrathoracic metastases (OR = 2.94, 95% CI: 1.08–7.69) were features independently associated with an increased risk of ICI-related pneumonitis (i.e., CIP) [50,51].

### 4.5. PD-1 Inhibitors

A meta-analysis of 19 trials conducted by Khunger M. et al. [52] demonstrated that PD-1 inhibitors were associated with a statistically significantly higher incidence of any-grade CIP compared to PD-L1 inhibitors (3.6% vs. 1.3%, *p* = 0.001). Furthermore, patients receiving PD-1 inhibitors experienced a higher incidence of Grade 3 or 4 pneumonitis (1.1% vs. 0.4%; *p* = 0.02) [52]. In a study by Fukihara et al. [25], the administration of pembrolizumab, as compared with nivolumab, was identified as an independent predictor of immune-related pneumonitis (i.e., CIP) [25].

### 4.6. Combination ICI Therapy

The incidence rate of CIP was higher in individuals receiving combination immune checkpoint inhibitor (ICI) therapy than in those receiving ICI monotherapy, regardless of tumor stage. Moreover, an inverse relationship was observed between cancer stage and CIP incidence in patients receiving combination ICI therapy; that is, patients with lower-stage cancer exhibited a higher rate of CIP. Tumor histologic type also influenced CIP development, with patients exhibiting the nonsquamous histologic type having a lower risk. Although female gender was associated with a higher incidence of CIP (incidence rate ratio [IRR] = 1.34; 95% CI: 0.67–2.66), this finding did not reach statistical significance [20].

### 4.7. Biomarkers

Hematological parameters and inflammatory markers have been identified as potential predictors of treatment response and prognosis in various cancers, including non-small-cell lung cancer (NSCLC), and they offer the advantages of being readily available, economical, and convenient [53]. Liu et al. [23] showed that lower pre-treatment hemoglobin and albumin levels were independent predictors of CIP [23]. Likewise, another study found that low serum albumin was associated with an increased risk of CIP and was an independent predictor of immune-related pneumonitis [25]. A study by Chao et al. [22] revealed that elevated baseline plasma IL-8 levels are associated with a decreased incidence of CIP, and previous ICI clinical trials demonstrated that greater pre-treatment IL-8 expression likely reflects an immunosuppressive, myeloid-enriched tumor microenvironment with limited adaptive T cell responses [22].

### 4.8. Baseline Pulmonary Function

Several analyses indicate that baseline pulmonary function may serve as a potential indicator of risk. Impaired lung function, as characterized by low FEV_1_ and reduced diffusion capacity prior to treatment, might predispose patients to CIP. Reuss et al. [45] reported that patients who developed CIP had a lower median FEV_1_% predicted at baseline than those without CIP, while baseline FVC% was similar [45]. In a prospective study by Suzuki et al. [46], poor baseline spirometry (reduced FVC and FEV_1_) and the presence of chronic dyspnea symptoms were significantly associated with the development of immune-related interstitial lung disease (i.e., CIP) during immunotherapy [46]. These findings suggest that underlying subclinical lung insufficiency or damage, such as from smoking or emphysema, may lower the threshold for triggering pneumonitis under ICI therapy [46].

Age ≥65 years and smoking history were associated with an increased risk of developing pneumonitis [26]. In an NSCLC-only series, the proportion of patients aged >70 years or those with prior ILD was higher in patients with ICI-related pneumonitis (i.e., CIP) than in those without (54.5% versus 30.3% and 18.2% versus 2.8%, respectively) [50,51].

Univariate analysis suggested that a history of chest radiotherapy was related to the incidence of CIP; however, multivariate logistic regression analysis showed that previous chest radiotherapy was not an independent risk factor for CIP [23].

Mao et al. [29], in a study of patients with NSCLC stages IIA–IIIB receiving neoadjuvant immunochemotherapy, it was revealed that post-neoadjuvant therapy fibrinogen level (post-FIB), post-neoadjuvant therapy diffusing capacity of the lungs for carbon monoxide expressed as a percentage of predicted (post-DLCO % pred), and d-value DLCO by alveolar volume (d-DLCO/VA % pred), as well as pre-neoadjuvant therapy BMI (pre-BMI), were independent risk factors for CIP. Thus, elevated BMI, increased fibrinogen levels, and decreased pulmonary diffusion function after neoadjuvant therapy are associated with an increased incidence of CIP [29].

### 4.9. Predictive Models

To improve risk stratification, researchers have proposed predictive models for CIP. Mao et al. [29], Chao et al. [22], and Lu et al. [56] developed predictive diagnostic models, or nomograms, that integrate various patient and disease factors to estimate an individual’s probability of developing pneumonitis during immunotherapy. These models typically incorporate factors such as lung function metrics, imaging findings, and patient demographics to generate a risk score. While these predictive models are promising, they require external validation and are not yet widely used in routine clinical practice.

**Figure 2:**
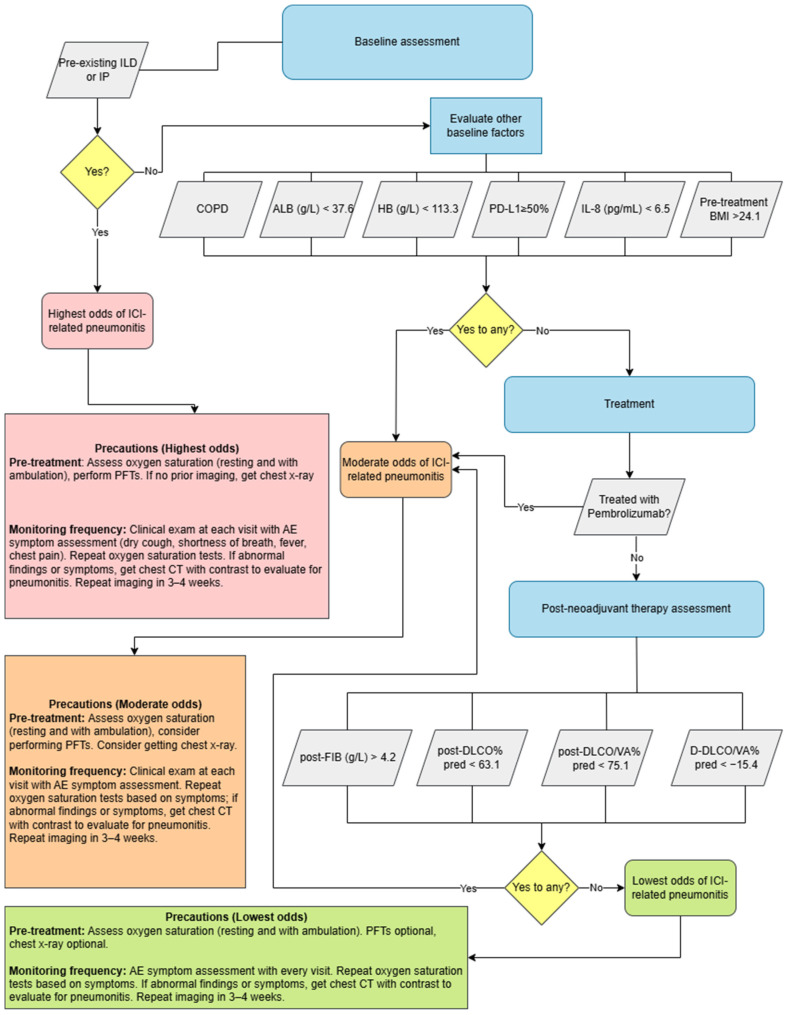
Clinical algorithm combining risk factors for immune checkpoint inhibitor (ICI) pneumonitis as identified in the literature with guidelines on management for immunotherapy-related pneumonitis from the NCCN [57]. Risk factors from Table 3b that have a *p* < 0.05 were included in this clinical algorithm. These include: pre-existing interstitial pneumonia (IP) [21]; pre-existing interstitial lung disease (ILD) [54]; chronic obstructive pulmonary disease (COPD) [22]; serum albumin level (ALB) [23,25]; hemoglobin (HB) [23]; PD-L1 expression [22]; IL-8 expression [22]; pre-treatment body mass index (BMI) [29]; treatment with pembrolizumab [25]; post-treatment fibrinogen levels (FIB) [29]; post-treatment diffusing capacity of the lung for carbon monoxide, expressed as a percentage of the predicted value (post-DLCO % pred) [29]; post-treatment diffusing capacity corrected for alveolar volume, expressed as a percentage of the predicted value (post-DLCO/VA % pred) [29]; and change in diffusing capacity per unit alveolar volume, expressed as a percentage of the predicted value (D-DLCO/VA% pred) [29]. ILD and IP have been combined into the same box to represent their overlapping definitions, though they are reported separately in the literature. Benchmarks for specific risk factors are the mean CIP group values as published in their respective studies. This figure was made using draw.io version 29.6.6; access link [https://app.diagrams.net], accessed on 24 March 2026.

In summary, a multitude of factors have been evaluated for association with CIP, and key risk factors have emerged. As stated, pre-existing ILD (strongly predictive of CIP), prior thoracic radiation, and possibly baseline pulmonary impairment all seem to confer higher risk. Traditional patient factors (age, smoking) and basic tumor characteristics have not shown strong or consistent correlations. Ongoing prospective studies and pooled analyses are needed to definitively determine which patients are most susceptible to pneumonitis so that monitoring and prophylactic strategies can be tailored accordingly. Figure 2 represents a clinical algorithm combining the above factors with current clinical guidelines for management of CIP [57], and Table 3 summarizes the statistical data behind these risk factors.

## 5. Discussion

A wide array of potential risk factors has been implicated in the development of ICI-related pneumonitis in NSCLC; however, data regarding their impact on CIP incidence remain somewhat inconsistent across studies. This inconsistency can be attributed to limitations inherent in many available studies, including small patient cohorts, retrospective designs with inherent biases, heterogeneous patient criteria, a lack of standardized and detailed pulmonary function assessments, and limited longitudinal follow-up. In particular, the precise role of pulmonary function tests (PFTs) in the early diagnosis and prediction of CIP remains unclear.

It is crucial to identify patients at elevated risk for CIP to enable vigilant monitoring and prompt intervention as soon as pneumonitis is suspected. Recent efforts have focused on developing nomograms that integrate multiple risk factors into an estimated probability of CIP, such as those proposed by Mao et al. [29], Chao et al. [22] and Lu et al. [56]. These tools, once validated, could assist clinicians in stratifying risk and personalizing immunotherapy approaches, such as implementing closer imaging surveillance or prophylactic measures for high-risk patients.

Clinicians should be particularly attentive to patients who exhibit one or multiple risk factors for CIP development, and should consider more frequent and attentive imaging, with more attention paid to telltale symptoms of CIP during treatment such as dry cough, shortness of breath, fever, and chest pain. In particular, clinicians should tend toward the more conservative and proactive end of published monitoring guidelines when considering high-risk patients, given the importance of early identification and treatment of this dangerous side-effect. At this time, there is no definitive evidence as to whether pembrolizumab should be foregone altogether for patients with significant risk factors for CIP; the data described in this review should instead prompt clinicians to proceed with caution throughout ICI treatment of high-risk patients. In such cases, an interdisciplinary approach involving the patient’s full circle of care should be used, including close following of the patient via nurses and family practitioners, and consultation with pulmonologists and thoracic radiologists as necessary [24]. Additional imaging and pulmonary function tests may serve to provide further insight into disease development or progression. The development of any symptoms of CIP by high-risk patients should be treated with the utmost gravity, as early detection is crucial in proper care for this life-threatening complication; as such, a team of vigilant practitioners closely following high-risk patients receiving ICIs is key to ensuring improved clinical outcomes over time. While official guidelines do exist for the management of CIP depending on patient risk factors and history [57], more specific guidance must be developed regarding appropriate monitoring protocols for patients identified as being at-risk for later development of CIP, with particular courses of action recommended based on patient risk factors. In particular, the role of PFTs in the prediction and identification of CIP must be elucidated, as they are a simple and non-invasive test that could prove invaluable in the monitoring of patients at risk of developing CIP if a correlation is found. As more is understood about the risk stratification of CIP, researchers must also be prepared and responsive in developing more granular and specific clinical guidelines for the monitoring and prophylaxis of at-risk patients, as well as risk-benefit stratification to support clinical decision-making around choice of ICIs.

Ultimately, further research is needed to refine our understanding of CIP. Future prospective studies with larger patient populations and standardized data collection are essential to confirm risk factors, to identify reliable biomarkers for early detection, including clarifying the role of PFTs, and to improve management strategies. Such studies will help ensure that the life-saving benefits of immunotherapy in NSCLC can be realized while minimizing the impact of serious toxicities like pneumonitis.

## Figures and Tables

**Table 1:** Incidence of pneumonitis in real-world practice.

Study	Year	Number of Patients	Patients with CIP (%)
Chao Y et al. [22]	2022	164	20 (12.2)
Liu X et al. [23]	2023	222	41 (18.5)
Altan M et al. [24]	2023	419	40 (9.5)
Fukihara J et al. [25]	2019	170	27 (16)
* Suresh K et al. [20]	2018	205	39 (19)
Sawa K et al. [26]	2023	328	37 (11.3)
Sumi T et al. [27]	2024	76	24 (31.6)
Yamaguchi T et al. [21]	2022	125	17 (13.6)
Zeng Z et al. [28]	2022	122	19 (15.5)
Mao Z et al. [29]	2024	245	28 (11.4)

Key: Table includes illustrative examples of reported incidence rates of ICI-related pneumonitis in real-world NSCLC patient cohorts (retrospective studies and pharmacovigilance analyses). Data are adapted from the literature; the study with the asterisk * by Suresh et al. [20] is a multi-center retrospective analysis (incidence 19% in an academic center cohort).

**Table 2:** Incidence of ICI-induced pneumonitis in NSCLC clinical trials.

Author	Study	Treatment Arm	Number of Patients That Received ICIs	Patients with CIP (%)
Cascone et al. [4]	CheckMate 77T	Nivolumab (neoadjuvant + adjuvant)	228	12 (5.3)
Wakelee et al. [30]	KEYNOTE-671	Pembrolizumab (neoadjuvant + adjuvant)	396	22 (5.6)
Heymach et al. [31]	AEGEAN	Durvalumab (neoadjuvant + adjuvant)	401	15 (3.7)
Reck et al. [32]	KEYNOTE-024	Pembrolizumab (first-line)	154	9 (5.8)
Herbst et al. [33]	IMpower110	Metastatic: First-line (atezolizumab monotherapy in high PD-L1 expressers)	286	11 (3.8)
Gandhi et al. [34]	KEYNOTE-189	Metastatic (nonsquamous): First-line pembrolizumab + chemotherapy	405	18 (4.4)
Socinski et al. [35]	IMpower150	Metastatic (nonsquamous): First-line atezolizumab + bevacizumab + chemotherapy	393	11 (2.8)
Nishio et al. [36]	IMpower132	Metastatic (nonsquamous): First-line atezolizumab + chemotherapy	291	18 (6.2)
Gogishvili et al. [37]	EMPOWER- Lung 3 *	Metastatic: First-line (cemiplimab + chemotherapy)	312	1 (0.3) #
Paz-Ares et al. [38]	CheckMate 9LA	Metastatic: First-line (combination nivolumab + ipilimumab plus limited-cycle chemotherapy)	358	21 (5.9)
Forde et al. [39]	CheckMate 816	Early-stage (resectable): Neoadjuvant nivolumab + chemotherapy before surgery	176	2 (1.1)
Garassino et al. [40]	PACIFIC-6	Unresectable: Durvalumab after sequential chemoradiotherapy	117	2 (1.7)
Paz-Ares et al. [41]	KEYNOTE-407	Metastatic: First line (pembrolizumab + chemotherapy)	278	18 (6.5)
Antonia et al. [42]	PACIFIC	Unresectable: Durvalumab after concurrent chemoradiotherapy	475	60 (12.6)

Key: Table summarizes pneumonitis incidence in NSCLC arms of selected immunotherapy clinical trials. For example, CheckMate 77T (perioperative nivolumab) reported a 5.3% incidence of pneumonitis [4], while KEYNOTE-671 (perioperative pembrolizumab) reported 5.6% [30]. Rates in purely metastatic trials (e.g., KEYNOTE-024 [32], IMpower110 [33]) were in the range of ~3–6%. These controlled trial rates are generally lower than those observed in real-world studies (see Table 1). * Treatment-emergent adverse events regardless of attribution: pneumonitis—13 (4.2), immune-mediated pneumonitis—1 (0.3); treatment-related AEs in the safety population: pneumonitis—12 (3.8), immune-mediated pneumonitis—1 (0.3); sponsor-identified immune-related adverse events (irAEs): pneumonitis—5 (1.6), immune-mediated pneumonitis—1 (0.3). # Immune-mediated pneumonitis.

**Table 3:** (**a**) Risk factors in the development of CIP and their respective hazard ratios. (**b**) Risk factors in the development of CIP and their respective odds ratios.

(**a**)								
**Study**	**Risk Factor**		**Univariate** **HR**		**CI**	**Multivariate** **HR**	**CI**	
Altan et al. [24]	ILD					5.35	(1.08, 26.55)	
	-overall		4.03		(0.97, 16.75)			
	-never smokers		26.91		(2.8, 258.96)			
	-ever smokers		2.21		(0.30, 16.25)			
	Baseline symptoms Shortness of breath					2.12	(1.05, 4.31)	
	-overall		1.95		(0.98, 3.91)			
	-never smokers		2.01		(0.59, 8.05)			
	-ever smokers		2.08		(0.92, 4.68)			
	Cough							
	-overall		1.73		(0.87, 3.42)			
	-never smokers		0.65		(0.13, 3.12)			
	-ever smokers		2.32		(1.04, 5.17)			
(**b**)								
**Study**		**Risk Factor**	**Univariate Analysis**			**Multivariate Analysis**		
			**OR**	**CI**	***p*** **-Value**	**OR**	**CI**	***p*** **-Value**
Liu et al. [23]		ALB (g/L)	0.909	0.870–0.950	<0.001 *	0.943	0.901–0.987	0.011 *
		HB (g/L)	0.976	0.965–0.987	<0.001 *	0.988	0.975–1.002	0.093
Fukihara et al. [25]		Drug, pembrolizumab	3.365	1.431–7.909	0.005 *	3.259	1.361–7.807	0.008
		Albumin (g/dL)	0.367	0.176–0.768	0.008 *	0.381	0.179–0.808	0.012 *
Suresh et al. [20]		Female Sex	1.12	(0.53–2.35)	0.75	0.38	(0.17–0.82)	0.01 *
		Adenocarcinoma	0.42	(0.19–0.89)	0.02 *			
		Combination ICI	1.72	(0.80–3.67)	0.16			
Sugano et al. [54]		Pre-existing IP No vs. yes	12.6	2.46–61.8	0.002 *	14.7	2.16–99.6	0.006 *
Yama-guchi et al. [21]		Pre-existing ILD Yes vs. no	14.00	(4.37–44.86)	<0.0001 *	19.07	(4.24–85.67)	0.0001 *
		Chemother-apy PEM vs. PTX/nab-PTX	2.49	(0.82–7.55)	0.11	5.67	(1.28–25.11)	0.022 *
Chao et al. [22]		COPD	3.460	(1.257–9.525)	0.016 *	7.194	(1.130–45.798)	0.037 *
		PD-L1 expression status ≥ 50%	3.211	(1.090–9.458)	0.034 *	7.184	(1.154–44.721)	0.035 *
		IL-8 (pg/mL)	0.831	(0.691–0.999)	0.049 *	0.758	(0.587–0.978)	0.033 *
Mao et al. [29]		Model 1				Mod.1		
		pre-BMI (kg/m^2^)	1.15	1.02–1.30	0.028 *	1.27	1.10–1.47	0.001 *
		post-FIB (g/L)	2.21	1.53–3.3	<0.001 *	2.09	1.43–3.24	<0.001 *
		post-DLCO % pred	0.95	0.92–0.98	0.001 *	0.94	0.91–0.98	0.001 *
		post-DLCO/VA % pred	0.95	0.93–0.98	<0.001 *			
		Model 2						
		pre-BMI (kg/m^2^)	1.15	1.02–1.30	0.028 *	1.19	1.04–1.37	0.013 *
		post-FIB (g/L)	2.21	1.53–3.35	<0.001 *	2.25	1.56–3.40	<0.001 *
		D-DLCO/VA % pred	0.96	0.93–0.98	0.001 *	0.96	0.93–0.99	0.003 *

Key: Table 3a: HRs of CIP development per risk factor as reported by Altan et al. [24]. HR (Hazard Ratio): The HR represents the divergence in event rate between two groups. For example, never-smokers with a cough before treatment developed CIP at a lower rate than the overall cohort of patients with a cough at baseline, whereas ever-smokers with a baseline cough developed CIP at a greater rate. Subgroup types are underlined (e.g., ILD and baseline symptoms) and subgroup data indicate that these analyses were performed separately for different patient cohorts (overall, never smokers, ever smokers). CI (Confidence Interval): The 95% CI provides the range within which the true HR is expected to lie with 95% certainty. Table 3b: ORs for various risk factors for CIP as reported in the literature. OR (Odds Ratio): ratio of events (patients developing CIP) to non-events (patients not developing CIP). For continuous variables (e.g., blood albumin levels in Liu et al. [23]), the OR represents the change in odds of developing CIP per one-unit change in the predictor variable (for example, CIP odds increase by 8.09% per each 1 g/L decrease in blood albumin level [23]). CI (Confidence Interval): The 95% CI provides the range within which the true OR is expected to lie with 95% certainty; *p*-value: Indicates the statistical significance of the association; A *p*-value less than 0.05 is generally considered statistically significant, and statistically significant *p*-values in Table 3b have been marked with asterisks (*) for clarity. Risk factors post-DLCO % pred and d-DLCO/VA% pred were split into two combinations for analysis to prevent multicolinearity and overfitting [29].

## Data Availability

No data were created or analyzed in this study. Data sharing is not applicable to this article.

## Abbreviations

The following abbreviations are used in this manuscript:

NSCLC	Non-Small Cell Lung Cancer
HR	Hazard Ratio
irAEs	Immune-related Adverse Events
OR	Odds Ratio
CIP	Checkpoint Inhibitor Pneumonitis
ICP	Immune Checkpoint Pneumonitis
ALB	Albumin
HB	Hemoglobin
ILD	Interstitial Lung Disease
IP	Interstitial Pneumonia
ICI	Immune Checkpoint Inhibitor
PD-L1	Programmed Death-Ligand 1
PEM	Pemetrexed
PTX	Paclitaxel
nab-PTX	Nanoparticle Albumin-Bound Paclitaxel
COPD	Chronic Obstructive Pulmonary Disease
IL-8	Interleukin-8
pre-BMI	Pre-treatment Body Mass Index
post-FIB	Post-treatment Fibrinogen Level
post-DLCO % pred	Post-treatment Diffusing Capacity of the Lung for Carbon Monoxide expressed as a percentage of the predicted value.
post-DLCO/VA % pred	Post-treatment DLCO (diffusing capacity) corrected for alveolar volume, expressed as a percentage of the predicted value.
D-DLCO/VA % pred	Change in DLCO/VA (diffusing capacity per unit alveolar volume) expressed as a percentage of the predicted value.
Mod.1	Model 1
Mod.2	Model 2
